# Molecular detection and subtype characterization of *Blastocystis* in diarrheal outpatients in Shanghai, China: A case-control study

**DOI:** 10.1371/journal.pntd.0014461

**Published:** 2026-06-26

**Authors:** Wei Zhao, Yuhan Guan, Qi Yu, Kuan Zhang, Zhongkai Zhang, Huanhuan Zhou, Bowen Song

**Affiliations:** 1 School of Basic Medical Sciences, Wenzhou Medical University, Wenzhou, China; 2 Department of Clinical Laboratory, Shanghai Pudong New Area People’s Hospital, Shanghai, China; 3 Laboratory Department, Qingdao South District Center for Disease Control and Prevention, Qingdao, Shandong, China; 4 School of Medical Humanities and Management, Wenzhou Medical University, Wenzhou, PR China; 5 Key Research Center of Philosophy and Social Sciences of Zhejiang Province (Institute of Medical Humanities, Wenzhou Medical University), Chashan University Town, Wenzhou, Zhejiang, PR China; Advanced Centre for Chronic and Rare Diseases, INDIA

## Abstract

**Background:**

*Blastocystis* is the most prevalent enteric protozoan colonizing the human gastrointestinal tract, yet its clinical relevance and molecular epidemiological profiles in Shanghai, China’s largest international megacity, remain unclear. The present study investigated the occurrence, subtype distribution, genetic diversity, and clinical associations of *Blastocystis* in diarrheal outpatients in Shanghai, China.

**Methods:**

A case-control study was conducted with 370 fecal specimens (220 from diarrheal patients, 150 age- and sex-matched non-diarrheal healthy controls) collected between June 2023 and March 2026. *Blastocystis* was detected via PCR amplification of the *small subunit ribosomal RNA* (*SSU rRNA*) gene, with Sanger sequencing for subtype assignment. Statistical analyses were performed using categorical tests and logistic regression.

**Results:**

The overall *Blastocystis* prevalence was 11.62% (43/370), significantly higher in diarrheal patients (16.8%; 37/220) than in controls (4.0%; 6/150, *P* < 0.001), with a higher detection rate in chronic (27.3%; 12/44) versus acute (14.2%; 25/176) diarrhea. Only two subtypes were identified: dominant ST3 (76.7%) and ST1 (23.3%). ST1 exhibited higher genetic diversity and showed an exploratory association with chronic diarrhea (*P* = 0.042, uncorrected for multiple testing). Age 7–18 years showed a borderline positive correlation with detection in diarrheal patients.

**Conclusions:**

This study found that *Blastocystis* detection was significantly correlated with diarrheal symptoms in the Shanghai outpatient population. ST3 was the dominant circulating subtype, and a potential association between ST1 and chronic diarrhea was identified, which requires confirmation in larger prospective studies. These results contribute to the regional molecular epidemiological database of *Blastocystis* and may inform clinical decision-making for patients with persistent unexplained diarrhea.

## Author summary

*Blastocystis* is one of the most common intestinal protozoa colonizing the human gastrointestinal tract worldwide, but its association with diarrhea remains controversial, and its molecular epidemiological characteristics in Shanghai, China’s largest international megacity, are poorly understood. We conducted a case-control study involving 370 participants, including 220 diarrheal outpatients and 150 age- and sex-matched healthy controls. We used PCR combined with Sanger sequencing to detect and subtype *Blastocystis*. We found that the detection rate of *Blastocystis* was significantly higher in diarrheal patients (16.8%) than in healthy controls (4.0%). Only two subtypes (ST1 and ST3) were identified, with ST3 being the dominant subtype (76.7%). ST1 showed higher genetic diversity and had an exploratory association with chronic diarrhea (uncorrected for multiple testing). Our findings provide important baseline data for clinical auxiliary screening and public health surveillance of *Blastocystis* in Shanghai, China.

## 1. Introduction

Infectious diarrhea remains a major and persistent global public health concern, caused by a broad spectrum of etiological agents including viruses, bacteria, and parasites [1]. However, routine clinical diagnostics and epidemiological surveillance for diarrheal diseases predominantly focus on common enteric viruses and bacteria. In contrast, *Blastocystis* and other intestinal protozoa are rarely included in standard clinical screening panels, resulting in a limited understanding of their prevalence, transmission dynamics, and clinical associations across different populations [2].

*Blastocystis* is the most prevalent enteric protozoan colonizing the human gastrointestinal tract worldwide, with primary transmission via the fecal-oral route [3]. The clinical significance of *Blastocystis* has long been controversial. Some studies have classified *Blastocystis* as a harmless commensal organism due to its frequent detection in asymptomatic individuals [4]. However, a large number of studies have reported a correlation between *Blastocystis* colonization and a variety of gastrointestinal symptoms, including acute and chronic diarrhea, irritable bowel syndrome (IBS), and extraintestinal manifestations such as urticaria [5,6]. At present, whether this correlation indicates a causal effect remains a core academic controversy, and no consensus has been reached to date [7]. Conventional light microscopy, the traditional diagnostic method for *Blastocystis*, has critical limitations: low analytical sensitivity, inability to resolve the parasite’s genetic diversity, and inconsistent identification across laboratories [8,9]. The unresolved clinical relevance debate and diagnostic limitations have led to the long-term neglect of *Blastocystis* in clinical settings and regional enteric disease surveillance systems.

Polymerase chain reaction (PCR) combined with *small subunit ribosomal RNA* (*SSU rRNA*) gene sequencing is the reference standard molecular method for *Blastocystis* detection and genotyping [10]. It has higher analytical sensitivity than conventional microscopy, reducing underdetection, and enables subtype classification, which is crucial for understanding the protozoan’s clinical correlation [9]. Genotyping studies suggest that the inconsistent clinical findings of *Blastocystis* may be related to differences in clinical correlation among different subtypes (STs), and some studies have explored potential phenotype differences among subtypes through in vitro and in vivo experiments [11]. For instance, ST7 has been consistently associated with diarrhea and intestinal inflammation in human cohort and murine model studies, while ST4 exhibits commensal-like characteristics in multiple preclinical models [12]. The global distribution of STs also shows pronounced geographical heterogeneity: ST3 is the most prevalent and globally distributed subtype in humans, whereas ST4 shows a highly restricted geographic distribution, being common in Europe but rare or absent in Africa, Asia, and South America [13].

Beyond resolving diagnostic limitations, *SSU rRNA* genotyping enables tracing of infection sources and characterization of transmission dynamics. A total of 46 *Blastocystis* subtypes (ST1-ST46) have been named to date and 38 of them are valid based on standard criteria [14]. Sixteen subtypes (ST1-ST10, ST12, ST14, ST16, ST23, ST35, and ST41) have been found in humans and ST1 to ST4 are the dominant ones accounting for more than 95% of human infections [14,15]. Different *Blastocystis* subtypes exhibit distinct host ranges, with ST1 showing broad host specificity among humans and various mammals, ST2 and ST3 occurring mainly in humans and non‑human primates, ST4 being common in rodents and humans in Europe, ST5 being highly specific to pigs and cattle, ST6 and ST7 occurring primarily in birds, and ST10 frequently infecting cattle, sheep and other ungulates [13,16]. These findings, combined with the subtype-related clinical heterogeneity of *Blastocystis*, highlight the public health need for molecular screening of *Blastocystis* in patients with persistent gastrointestinal symptoms.

Against this backdrop, molecular epidemiological studies of *Blastocystis* have been implemented across multiple provinces in China [17–19]. These investigations have revealed considerable variation in human infection rates, distinct geographic patterns of subtype distribution, and widespread occurrence in both domestic and wild animal reservoirs [17–19]. However, the available epidemiological data remain geographically fragmented, and systematic investigations into the clinical features and zoonotic risk of *Blastocystis* in large, highly mobile urban populations remain lacking. As China’s leading international megacity, Shanghai has high population density, intense cross-regional and cross-border population mobility, and dense urban environments—all factors known to facilitate enteric pathogen transmission and elevate public health risks. To date, few comprehensive molecular epidemiological studies have characterized *Blastocystis* prevalence, subtype distribution, and clinical associations in diarrheal patients in Shanghai [20,21], leaving a critical gap in local enteric disease surveillance and control. Given these regional epidemiological discrepancies and unresolved subtype-specific clinical relevance, we hypothesized that *Blastocystis* detection rates would be significantly higher in diarrheal outpatients than in healthy controls, and that specific *Blastocystis* subtypes would be associated with distinct clinical presentations, particularly chronic diarrhea. To test this hypothesis, we conducted a case-control study to define the prevalence of *Blastocystis* in diarrheal and healthy populations in Shanghai, characterize the subtype composition and genetic diversity of local isolates, and explore its clinical correlations with gastrointestinal symptoms.

## 2. Materials and methods

### 2.1 Ethical statement

The protocol was reviewed and approved by the Ethics Committee of Wenzhou Medical University (Approval No. SCILLSC-2023-01). Before sample collection, written informed consent was obtained from all enrolled participants or their legal guardians for those under 18 years old. For 7–18-year-old minors, both guardian and subject written informed consents were obtained. All participant data and fecal specimens were anonymized with unique alphanumeric identifiers for confidentiality, and clinical data were de-identified before statistical analysis.

### 2.2 Study population and fecal specimen collection

A case-control study was conducted with 370 fecal specimens collected from eligible participants recruited at a tertiary hospital in Shanghai, China, between June 2023 and March 2026. The cohort comprised 220 specimens from patients with clinically diagnosed acute or chronic diarrhea (case group) and 150 age- and sex-frequency matched non-diarrheal healthy individuals (control group). In the control group, 73 individuals were from the same hospital’s physical examination center, and 77 (all < 18 years old) were from 4 schools in the same residential area as the hospital. Sample size calculation was not performed prior to the study initiation due to the exploratory and observational design of this investigation.

Inclusion criteria for the case group were defined in accordance with World Health Organization (WHO) standards (World Health Organization, 2024) [22]: (1) presentation with ≥ 3 loose or watery bowel movements within a 24-hour period; (2) no administration of antibiotics, antiparasitic agents, or probiotics within four weeks prior to specimen collection. Only patients with acute and chronic diarrhea were enrolled. Acute diarrhea was defined as symptom duration < 14 days, and chronic diarrhea as symptom duration ≥ 4 weeks. Exclusion criteria included a history of chronic inflammatory bowel disease, immunocompromising conditions, or gastrointestinal surgery within six months prior to enrollment.

Inclusion criteria for the control group were: (1) absence of diarrhea, abdominal discomfort, or other gastrointestinal symptoms within three months prior to sampling; (2) no history of chronic gastrointestinal disease or immunodeficiency; (3) no exposure to antimicrobial agents within four weeks before specimen collection.

Fresh fecal specimens (≥ 2 g per participant) were collected into sterile, DNase/RNase-free, screw-capped fecal collection containers, labeled with unique anonymized identifiers, and immediately stored at 4 °C pending transportation.

### 2.3 Specimen transportation and storage

All specimens were maintained at a constant 4 °C during transportation, and delivered to the Parasitology Laboratory of Wenzhou Medical University via validated temperature-controlled cold chain logistics within 24 hours of collection. Upon receipt, specimens were aseptically homogenized, divided into aliquots, and stored at −80 °C until genomic DNA extraction. No repeated freeze-thaw cycles were performed prior to experimental analysis.

### 2.4 Genomic DNA extraction

Total genomic DNA was extracted from 200 mg of each homogenized fecal specimen using the QIAamp fast DNA Stool Mini Kit (QIAGEN, Hilden, Germany), following the manufacturer’s standard protocol with an optimized extended lysis step (95 °C for 10 min) to maximize the recovery of protozoan genomic DNA [23]. The concentration and purity of extracted DNA were quantified using a NanoDrop 200 UV-Vis spectrophotometer (Thermo Fisher Scientific, Wilmington, DE, USA). DNA specimens with an A260/A280 ratio of 1.8–2.0 were deemed eligible for downstream PCR amplification, and eligible extracts were stored at −20 °C until use.

### 2.5 PCR amplification for *Blastocystis* detection

*Blastocystis* was detected via PCR amplification of the hypervariable barcode region of the *SSU rRNA* gene, using the universal primer set BhRD1 (5′-GGA GGT AGT GAC AAT AAA TC-3′) and BhRD2 (5′-TGC TTT CGC ACT TGT TCA TC-3′), which amplifies a ~ 500 bp fragment [24]. PCR reactions were performed in a final volume of 25 μL, consisting of 2.5 μL of 10 × PCR buffer, 2 μL of dNTP mix (2.5 mM each), 0.5 μL of forward and reverse primers (10 μM each), 0.5 μL of TaKaRa Taq DNA Polymerase (5 U/μL; TaKaRa Bio Inc., Tokyo, Japan, Cat. No. R500Z), 2 μL of template DNA, and 17 μL of nuclease-free water. Thermal cycling conditions were as follows: initial denaturation at 94 °C for 5 min; 35 cycles of denaturation at 94 °C for 30 s, annealing at 55 °C for 30 s, and extension at 72 °C for 1 min; and a final extension at 72 °C for 10 min.

To prevent amplicon contamination, pre- and post-PCR procedures were conducted in physically separated biosafety cabinets, with dedicated pipettes and disposable consumables. Each PCR run included a sequence-validated *Blastocystis* positive control (genomic DNA from goat-derived ST10) and a no-template negative control (nuclease-free water) to validate amplification reliability [25]. PCR amplicons were separated via 1.5% (w/v) agarose gel electrophoresis stained with GelRed nucleic acid stain (Biotium Inc., Hayward, CA, USA), and visualized using a ChemiDoc XRS + gel imaging system (Bio-Rad Laboratories, Hercules, CA, USA). Specimens yielding a single distinct band at the expected 500 bp size were classified as *Blastocystis*-positive.

### 2.6 Sanger sequencing and subtype assignment

All positive PCR amplicons were purified using the QIAamp PCR Purification Kit (Qiagen, Hilden, Germany), and bidirectional Sanger sequencing was performed with the BhRD1/BhRD2 primer set at Sangon Biotech Co., Ltd. (Shanghai, China). Raw sequencing reads were quality-trimmed, edited, and assembled into consensus sequences using SeqMan Pro software (DNASTAR Inc., Madison, WI, USA), with low-quality bases (Phred score < 20) removed from the 5′ and 3′ ends. Only sequences with a valid read length ≥450 bp were retained for downstream analysis.

Species identification was confirmed via BLASTn (https://blast.ncbi.nlm.nih.gov/Blast.cgi) alignment against the NCBI GenBank database, with a threshold of ≥ 98% nucleotide identity, ≥ 95% query coverage, and E-value ≤ 1e-10 for valid *Blastocystis* identification [10]. Sequences with 100% nucleotide identity to a reference allele in the PubMLST database (https://pubmlst.org/blastocystis/) were assigned to the corresponding subtype and allele number; sequences with no exact match but ≥ 97% identity to a single reference subtype were classified into the closest matching subtype [10]. All unique representative sequences generated in this study have been deposited in NCBI GenBank under accession numbers: PZ256387 to PZ256399.

### 2.7 Genetic diversity and Phylogenetic analysis

Genetic diversity analysis was performed using DnaSP 6.12.03 software [26]. Standardized indices including nucleotide diversity (π), Watterson’s θ, and segregating sites per site were calculated to correct for differences in sample size and sequence length. Representative sequences capturing all unique haplotypes were used for analysis to avoid bias from redundant identical sequences.

Phylogenetic analysis was conducted using MEGA12 software (https://www.megasoftware.net/). The obtained *Blastocystis SSU rRNA* gene sequences and reference sequences of known *Blastocystis* STs from PubMLST database were aligned with the ClustalW algorithm. A neighbor-joining (NJ) tree was constructed using the Kimura 2-parameter (K2) model, with bootstrap support evaluated by 1000 replicates (only values ≥ 50 were shown). *Proteromonas* sp. (GenBank accession no. U37108) was used as the outgroup to root the tree.

### 2.8 Statistical analysis

All analyses were performed using IBM SPSS Statistics 26.0 (IBM Corp., Armonk, NY, USA). Categorical variables were reported as counts (proportions), with between-group comparisons using Pearson’s χ² test or Fisher’s exact test (for expected cell counts < 5).

Univariate logistic regression was used to identify factors correlated with *Blastocystis* detection. Multivariate logistic regression was only conducted in the diarrheal case group (37 positive events), including variables with *P* < 0.10 in univariate analysis plus age, sex, and residential region. The control group (only 6 positive events) did not meet regression sample size requirements, so only descriptive univariate analysis was performed. A two-sided *P* < 0.05 was considered statistically significant. Multiple testing correction was not performed due to the exploratory nature of this study; results should be interpreted with caution.

Post hoc statistical power analysis was performed using G*Power 3.1 software to evaluate the reliability of our statistical tests [27]. For the primary case-control comparison of *Blastocystis* prevalence, the achieved power was 96.8% (α = 0.05, two-sided) given the observed prevalence rates (16.8% in cases vs 4.0% in controls) and total sample size of 370. For the exploratory ST1-chronic diarrhea subgroup comparison, the achieved power was 62.3% (α = 0.05, two-sided) based on the observed proportions (66.7% in ST1-positive cases vs 21.4% in ST3-positive cases) and 37 total positive isolates. These results confirm that the primary analysis had adequate statistical power, while the subgroup analysis had moderate power and requires validation in larger prospective cohorts.

## 3. Results

### 3.1 Study cohort baseline characteristics and overall *Blastocystis* detection rate

A total of 370 participants were enrolled, including 220 diarrhea patients (case group) and 150 non-diarrheal healthy individuals (control group). Among cases, 176 had acute diarrhea and 44 had chronic diarrhea. Baseline analysis showed no significant differences in age, sex, and residential region between the two groups, indicating effective matching and satisfactory comparability (Table 1).

**Table 1:** Baseline characteristics of the study cohort and stratified *Blastocystis* detection rate.

Characteristic	Non-diarrheal Control Group (n = 150) n (%)	Total Diarrheal Case Group (n = 220) n (%)	Acute Diarrhea Subgroup (n = 176) n (%)	Chronic Diarrhea Subgroup (n = 44) n (%)	Between-group *P* value	*Blastocystis* Positive n/N (%)
**Age group**					0.976	
<6 years	57 (38.0)	88 (40.0)	72 (40.9)	16 (36.4)		16/145 (11.0)
7–18 years	24 (16.0)	35 (15.9)	26 (14.8)	9 (20.5)		11/59 (18.6)
19–60 years	54 (36.0)	75 (34.1)	59 (33.5)	16 (36.4)		11/129 (8.5)
>60 years	15 (10.0)	22 (10.0)	19 (10.8)	3 (6.8)		5/37 (13.5)
**Sex**					0.832	
Male	68 (45.3)	117 (53.2)	94 (53.4)	23 (52.3)		18/185 (9.7)
Female	82 (54.7)	103 (46.8)	82 (46.6)	21 (47.7)		25/185 (13.5)
**Residential region**					0.204	
Urban	12 (8.0)	31 (14.1)	24 (13.6)	7 (15.9)		7/43 (16.3)
Suburban	138 (92.0)	189 (85.9)	152 (86.4)	37 (84.1)		36/327 (11.0)
**Overall** *Blastocystis* **detection**	6/150 (4.0)	37/220 (16.8)	25/176 (14.2)	12/44 (27.3)	< 0.001	43/370 (11.6)

Abbreviations: n, number; %, percentage. Between-group *P* value was used to verify the balance of baseline characteristics between the control group and total diarrheal case group. *P* > 0.05 indicates no statistically significant difference in baseline characteristics between groups.

The overall *Blastocystis* detection rate in the cohort was 11.6% (43/370). The rate in the case group (16.8%, 37/220) was significantly higher than that in the control group (4.0%, 6/150) (χ^2^ = 14.27, df = 1, *P* < 0.001). Furthermore, the detection rate in the chronic diarrhea subgroup (27.3%, 12/44) was nearly twice that in the acute diarrhea subgroup (14.2%, 25/176) (χ^2^ = 4.3, df = 1, *P* = 0.038), and both subgroups had significantly higher rates than the control group (χ^2^ = 19.2, df = 1, *P* < 0.001 for chronic diarrhea vs control, χ^2^ = 9.8, df = 1, *P* = 0.002 for acute diarrhea vs control). By demographic characteristics, the highest detection rate was in 7–18-year-olds (18.64%), followed by urban residents (16.28%), and the lowest was in 19–60-year-olds (8.53%). There was no significant difference in detection rate across age, sex, or residential region in the total cohort (Table 1).

### 3.2 Factors correlated with *Blastocystis* detection

Univariate logistic regression of the total cohort identified factors correlated with *Blastocystis* detection. Acute and chronic diarrhea were significantly correlated with increased detection. Age 7–18 years showed a borderline positive correlation. No significant correlations were found between sex, residential region and detection (S1 Table). Multivariate logistic regression was conducted in the diarrheal case group. Variables with *P* < 0.10 in univariate analysis and relevant covariates (sex and residential region), even though *P* > 0.10, were included. After adjustment, age 7–18 years showed a borderline positive correlation in diarrheal patients, and no other demographic factors were independently correlated (Table 2). For the non-diarrheal control group, only six positive events were detected, so only descriptive univariate analysis was performed, and no significant correlations were observed (S2 Table). Overall, diarrheal clinical status was the factor most strongly correlated with *Blastocystis* detection in this cohort, while demographic characteristics showed limited independent correlation with detection.

**Table 2:** Multivariate logistic regression analysis of factors independently correlated with *Blastocystis* detection in diarrheal patients (n = 220).

Factors	Subgroup	Positive n/N (%)	Adjusted OR (95%CI)	*P* value
**Age group**	19–60 years (Ref)	9/75 (12.0)	1.00	–
	<6 years	14/88 (15.9)	1.21 (0.52–2.82)	0.657
	7–18 years	10/35 (28.6)	2.59 (0.94–7.14)	0.066
	>60 years	4/22 (18.2)	1.46 (0.40–5.30)	0.562
**Sex**	Male (Ref)	17/117 (14.5)	1.00	–
	Female	20/103 (19.4)	1.42 (0.68–2.97)	0.353
**Residential region**	Urban (Ref)	6/31 (19.4)	1.00	–
	Suburban	31/189 (16.4)	0.86 (0.32–2.32)	0.765

Abbreviations: Ref, reference group; OR, odds ratio; CI, confidence interval. The multivariate model was adjusted for age group, sex, and residential region.

### 3.3 Subtype distribution, clinical correlation, and genetic polymorphism

All 43 PCR-positive amplicons were sequenced bidirectionally, and two STs were identified (Table 3). ST3 was the predominant subtype (76.7%, 33/43), followed by ST1 (23.3%, 10/43). Among 37 *Blastocystis*-positive isolates from the diarrheal case group, ST3 accounted for 75.7% (28/37) and ST1 for 24.3% (9/37). Among the six positive isolates from the non-diarrheal control group, ST3 was dominant (83.3%, 5/6), and only one ST1 isolate (16.7%, 1/6) was detected, which was from a 37-year-old female suburban resident.

**Table 3:** Association of *Blastocystis* subtype distribution with demographic and clinical characteristics in case and control groups.

Characteristic	Subgroup	Case Group			Control Group	
		ST1 (n = 9) n	ST3 (n = 28) n	*P* value	ST1 (n = 1) n	ST3 (n = 5) n
Age group	<6 years	3	11	0.714	0	2
	7–18 years	2	8		0	1
	19–60 years	2	7		1	1
	>60 years	2	2		0	1
Sex	Male	5	12	0.721	0	2
	Female	4	16		1	3
Residential area	Urban	3	3	0.114	0	1
	Suburban	6	25		1	4
Diarrhea type	Acute diarrhea	3	22	0.042	–	–
	Chronic diarrhea	6	6		–	–

Abbreviations: n, number. All *P* values were calculated using two-tailed Fisher’s exact test. Due to the limited number of positive samples (n = 6) in the control group, statistical comparisons between groups were not performed. No statistically significant association was observed between subtype distribution and demographic characteristics in the case group (all *P* > 0.05), except for the significant association between ST1 detection and chronic diarrhea in diarrheal patients (*P* = 0.042). Multiple testing correction was not performed; the result should be interpreted with caution.

The correlation between subtype distribution and clinical phenotypes was further analyzed. Results showed that the proportion of chronic diarrhea was significantly higher in ST1-positive patients (66.7%, 6/9) than in ST3-positive ones (21.4%, 6/28; Fisher’s exact test, *P* = 0.042, uncorrected for multiple testing, exploratory finding, Table 3).

Sequence polymorphism analysis revealed significantly higher intrasubtype genetic diversity in ST1 isolates compared to ST3 isolates. Standardized genetic diversity indices were calculated to correct for differences in sample size and sequence length. The results showed that ST1 isolates had a nucleotide diversity (π) of 0.0136 and Watterson’s θ of 0.0245, while ST3 isolates had a π of 0.0037 and θ of 0.0031. This corresponds to 29 segregating sites in 8 ST1 representative sequences (0.0635 sites per base) versus 3 segregating sites in 5 ST3 representative sequences (0.0065 sites per base) (Table 4). The intrasubtype genetic diversity in ST1 isolates (29 SNPs) and ST3 isolates (three SNPs) is shown in Tables 5 and 6 (Tables 5 and 6).

**Table 4:** Standardized genetic diversity indices of *Blastocystis* ST1 and ST3 isolates based on representative sequences.

Subtype	GenBank accession numbers	Number of sequences (n)	Alignment length (bp)	Number of segregating sites (S)	Segregating sites per site (S/L)	Nucleotide diversity (π)	Watterson’s θ (θ_W_)
ST3	PZ256387–PZ256391	5	461	3	0.0065	0.0037	0.0031
ST1	PZ256392–PZ256399	8	457	29	0.0635	0.0136	0.0245

**Table 5:** Nucleotide variations at 3 polymorphic sites in five representative *Blastocystis* ST3 sequences.

GenBank accession number	173	213	270
PZ256387	A	T	C
PZ256388	G	C	C
PZ256389	A	T	T
PZ256390	A	T	G
PZ256391	A	C	C

**Table 6:** Nucleotide variations at 29 polymorphic sites in eight representative *Blastocystis* ST1 sequences.

GenBank accession number	16	21	25	26	175	181	186	187	188	198	199	200	203	204	205	206	208	209	210	211	212	213	246	257	270	305	324	354	365
PZ256392	G	A	C	A	A	G	T	G	A	C	T	C	G	A	C	A	T	A	A	G	T	C	C	T	C	G	A	A	T
PZ256393	C	C	A	C	G	G	A	A	T	T	A	G	A	G	A	T	A	T	C	A	C	T	G	T	G	C	G	T	G
PZ256394	C	C	C	A	A	A	T	G	A	C	T	C	G	A	T	A	T	A	A	G	T	C	G	T	C	C	G	T	G
PZ256395	C	C	C	A	A	G	T	G	A	C	T	C	G	A	C	A	T	A	A	G	T	C	G	T	C	C	G	T	G
PZ256396	C	C	A	C	A	G	T	G	A	C	T	C	G	A	C	A	T	A	A	G	T	C	G	T	C	C	G	T	G
PZ256397	C	C	C	A	A	G	T	G	A	C	T	C	G	A	C	A	T	A	A	G	T	C	G	T	G	C	G	T	G
PZ256398	C	C	C	A	A	G	T	G	A	C	T	C	G	A	C	A	T	A	A	G	T	C	G	C	C	C	G	T	G
PZ256399	C	C	C	C	A	G	T	G	A	C	T	C	G	A	T	A	T	A	A	G	T	C	C	C	C	C	G	T	G

The 33 ST3 isolates were clustered into five representative sequences (with an isolate count of 26, three, two, one, and one for each sequence, respectively). These sequences were assigned to two PubMLST alleles: allele 34 (n = 29), including three sequences identical to the reference sequence AB107965 and 26 sequences with one SNP variation; and allele 36 (n = 4), including two sequences identical to the reference sequence AB091234, one sequence with one SNP variation, and one sequence with two SNP variations (Table 7).

**Table 7:** Nucleotide variations and allele typing of representative *Blastocystis* sequences.

GenBank number (n)	ST	Allele No.	Number of polymorphic sites	Reference sequence	Nucleotide variation (site number)
PZ256387 (2)	ST3	Allele 36	0	AB091234	None
PZ256388 (1)	ST3	Allele 36	2	AB091234	A → G (173); T → C (213)
PZ256389 (26)	ST3	Allele 34	1	AB107965	G → T (270)
PZ256390 (3)	ST3	Allele 34	0	AB107965	None
PZ256391 (1)	ST3	Allele 36	1	AB091234	T → C (213)
PZ256392 (1)	ST1	Allele 88	8	AB070989	C → G (16); C → A (21); G → C (245); G → C (269); C → G (304); G → A (323); T → A (353); G → T (364)
PZ256393 (1)	ST1	Allele 2	10	AB107968	T → A (25); C → T (179); T → G (180); C → A (185); G → A (186); A → G (188); G → A (198); T → G (199); A → C (201); C → T (256)
PZ256394 (1)	ST1	Allele 88	3	AB070989	G → A (181); C → T (204); G → C (269)
PZ256395 (2)	ST1	Allele 88	1	AB070989	G → C (269)
PZ256396 (1)	ST1	Allele 88	3	AB070989	C → A (25); A → C (26); G → C (269)
PZ256397 (2)	ST1	Allele 88	0	AB070989	None
PZ256398 (1)	ST1	Allele 4	0	U51151	None
PZ256399 (1)	ST1	Allele 4	3	U51151	A → C (26); C → T (204); G → C (245)

For the 10 ST1 isolates, eight representative sequences were identified, which were divided into three PubMLST alleles: allele 2 (n = 1), which differed from the reference sequence AB107968 by 10 SNPs; allele 4 (n = 2), including one sequence fully matching the reference sequence U51151 and one sequence with three SNP variations; and allele 88 (n = 7), including two sequences identical to the reference sequence AB070989, with the remaining five sequences showing one to eight SNP variations from the reference (Table 7).

### 3.4 Phylogenetic analysis

**Fig 1:**
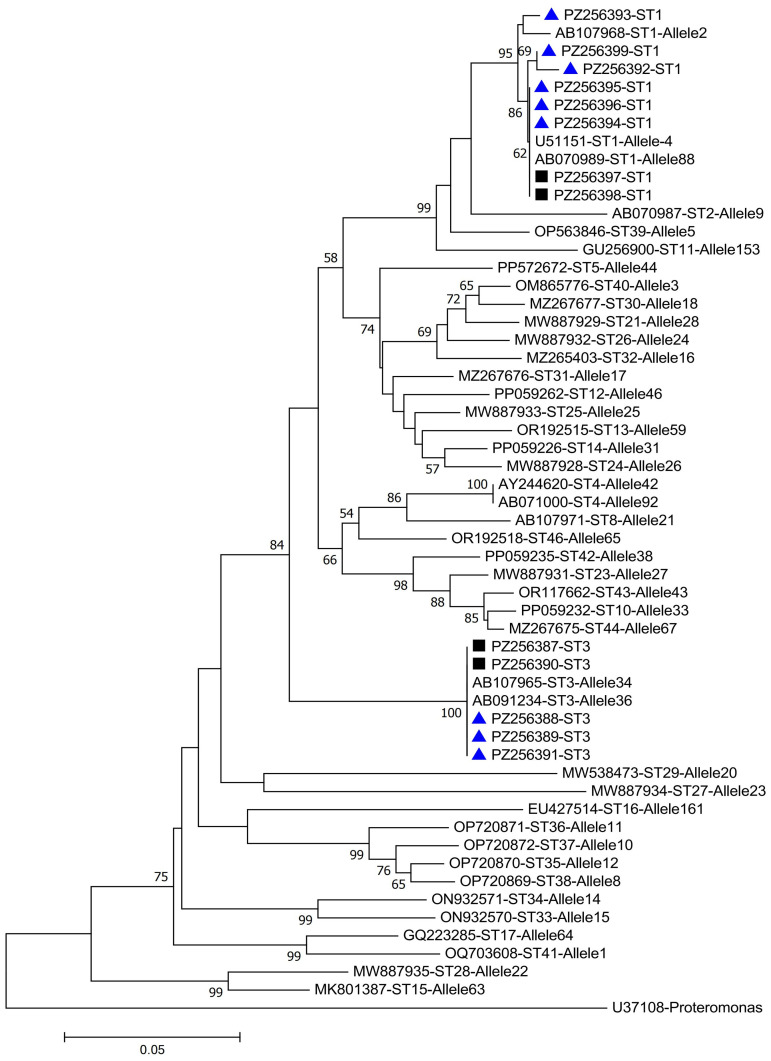
Phylogenetic tree of *Blastocystis* sp. based on the *SSU rRNA* gene. The tree was constructed by the Neighbor-Joining (NJ) method with the Kimura 2-parameter (K2) model, with bootstrap support estimated by 1000 replicates (only values ≥50 are shown). The scale bar (0.05) represents nucleotide substitutions per site. Blue filled triangles (▲) and black filled squares (■) represent novel and known sequences of *Blastocystis* STs obtained in this study, respectively. Outgroup: *Proteromonas* sp. (accession no. U37108).

All 13 representative *Blastocystis* sequences obtained from diarrhea patients in this study were classified into two subtypes: eight sequences belonged to ST1 and five sequences belonged to ST3. Each group of novel sequences clustered closely with the corresponding known reference sequences of the same subtype, with reliable bootstrap support (≥ 50). Reference sequences of other distinct *Blastocystis* subtypes formed separate clades. The tree was properly rooted with *Proteromonas* sp. (accession no. U37108) as the outgroup, with a stable topological structure (Fig 1).

## 4. Discussion

Globally, the association between *Blastocystis* sp. infection and diarrhea remains epidemiologically debated, with inconsistent findings across regions. In our cohort, the *Blastocystis* detection rate was significantly higher in diarrheal individuals (16.8%) than in non‑diarrheal controls (4.0%), with an adjusted OR of 4.78 (95% CI: 1.92–11.91, *P* < 0.001). Our finding of significantly higher *Blastocystis* detection rates in diarrheal outpatients is consistent with case-control studies targeting similar patient populations: a pediatric study from Wenzhou, China reported an 8.8% prevalence in children with acute or chronic diarrhea versus 2.0% in age-matched asymptomatic healthy controls [28]. Associations between *Blastocystis* colonization and gastrointestinal symptoms have also been documented in other distinct clinical populations: a study from North India found a 33.3% prevalence in patients IBS compared to 15.0% in healthy community controls [29]; a separate investigation reported a 22.8% detection rate in cancer patients with gastrointestinal symptoms versus 9.5% in asymptomatic cancer-free controls [30]. In contrast, several studies from different geographic regions have observed higher prevalence in asymptomatic individuals, supporting a potential commensal role for some *Blastocystis* subtypes: these include studies from Iran (29.4% in symptomatic gastrointestinal patients vs. 70.6% in asymptomatic controls) [31] and Korea (3.1% vs. 18.0%) [32]. These discrepancies may be attributed to differences in geographic subtype distribution, host immune status, detection methodologies, and study design. As epidemiological evidence indicates correlation rather than causation, further prospective and functional studies are needed to clarify the pathogenic potential of specific *Blastocystis* subtypes.

The prevalence of *Blastocystis* in Chinese human populations shows remarkable geographical heterogeneity, with reported rates ranging from 2.4% (Heilongjiang) to 43.3% (Guangxi) in general population studies [33,34]. However, only a small number of studies focused on diarrheal populations: the 16.8% infection rate in our Shanghai diarrheal cohort is higher than the 2.0% reported in Ningbo (Zhejiang) diarrhea outpatients [35] and 3.1% in Henan hospital patients with diarrhea [36], while comparable to the 8.8% in Wenzhou (Zhejiang) diarrheal children [23], 4.9% in Guangdong diarrhea outpatients [19] and 7.9% in Guizhou hospital patients [37]. Numerous factors contribute to the discrepancies in reported infection rates, making it difficult to determine the true prevalence of *Blastocystis* in Chinese diarrheal populations, and further large-scale multi-center epidemiological investigations specifically targeting diarrheal outpatients are warranted.

Our age-stratified analyses revealed that the significant correlation between *Blastocystis* detection and diarrhea was restricted to children and adolescents aged < 18 years, with the highest rate recorded in the 7–18 years subgroup. This age-dependent colonization pattern is consistent with previous studies reporting peak prevalence in 10–17-year-olds [20], and has been cross-validated by large-scale epidemiological studies from Nigeria, Brazil, Libya and other countries [38–40]. The elevated detection rate in school-age populations is primarily attributed to increased fecal-oral transmission exposure in campus aggregated settings, coupled with an incompletely mature intestinal mucosal immune barrier and a gut microenvironment permissive to *Blastocystis* colonization [4]. No significant between-group (diarrhea vs control) difference in prevalence was observed in adults aged ≥ 19 years, suggesting that the clinical correlation of *Blastocystis* detection is more pronounced in pediatric populations in Shanghai.

Unlike most previous studies reporting higher *Blastocystis* detection rates in rural/suburban populations than in urban areas [41], we observed lower rates in suburban areas relative to urban areas in both diarrheal and non-diarrheal groups. This discrepancy is largely explained by the unique urban-suburban structure of Shanghai. First, unlike typical rural regions with extensive livestock breeding, suburban Shanghai has limited animal husbandry and reduced human-livestock contact, lowering potential zoonotic exposure. Second, the extremely high population density, crowded public spaces, and frequent close interpersonal contact in urban Shanghai greatly facilitate fecal-oral transmission of *Blastocystis*, whereas more spacious living conditions and fewer high-intensity transmission scenarios in suburban areas reduce exposure risk. These findings highlight the impact of local socioeconomic conditions, breeding industry distribution, and population activity characteristics on *Blastocystis* transmission.

No significant correlation was observed between gender and *Blastocystis* detection in our study, which aligns with most global epidemiological findings. We only detected a non-significant trend of higher prevalence in females, which may be related to increased fecal-oral exposure from household chores, childcare, and close-contact activities, as well as gender-related differences in hygiene practices and social behavior [19].

Only two *Blastocystis* subtypes (ST1 and ST3) were identified in this study, with ST3 as the dominant subtype, consistent with the nationwide profile in China where ST3 and ST1 are the top two prevalent subtypes [19]. However, our study showed a restricted subtype repertoire, while previous studies across China (including an earlier Shanghai study) have reported a broader diversity of subtypes such as ST2, ST4, ST6 and ST7 [19,20]. This discrepancy may be attributed to the exclusive enrollment of diarrheal patients in our study, limited animal exposure in urban or suburban Shanghai, and the limited number of positive isolates reducing low-abundance subtype detection. The absence of Europe-dominant ST4 (rare in China) in our study further supports the intercontinental heterogeneity of subtype distribution, which collectively indicates that *Blastocystis* subtype distribution may be influenced by geographical factors [13,15].

Host tropism analysis offers key clues for inferring potential *Blastocystis* transmission routes in Shanghai. ST3’s restricted host tropism (nearly confined to humans and non-human primates, with a low probability of zoonotic potential, although it has occasionally been identified in other animals) [42] and its dominance in our cohort strongly suggest that human-to-human transmission may be the main spread route in urban and suburban of Shanghai, China. Conversely, ST1 is recognized as a typical generalist with an extremely broad host range. It infects humans, non-human primates, livestock, rodents, wildlife, and birds globally, which implies potential zoonotic risk [13]. Livestock exposure may be an important potential source of local ST1 detection, guiding targeted traceability and prevention. The identification of ST1 lineages with putative zoonotic risk also underscores the need for One Health-based *Blastocystis* surveillance, integrating human, animal and environmental sampling to further clarify transmission dynamics and reduce potential regional zoonotic risks.

We observed markedly higher intrasubtype genetic heterogeneity in *Blastocystis* ST1 isolates relative to ST3, consistent with previous global epidemiological studies [43]. This disparity is driven by two core factors. First, ST1’s broad host range enables frequent cross-species transmission between humans and animal reservoirs, fueling adaptive genetic evolution and polymorphism accumulation [13,43]. By contrast, ST3’s narrow host specificity, with transmission largely restricted to human populations, results in a more conserved genomic profile [42]. Second, the long-term endemicity of ST3 in human populations has led to founder effects and clonal expansion of dominant lineages, reducing its genetic diversity, while ST1 has multiple independent introduction routes from animal reservoirs, giving rise to more diverse circulating lineages in humans [43].

The high genetic heterogeneity of ST1 observed here carries important research implications. Extensive studies have explored phenotypic differences across *Blastocystis* subtypes and even within-subtype lineages via in vitro and in vivo experiments, yet no unified conclusion on its clinical significance has been reached [29,44]. In this study, 90% of ST1 isolates were detected in diarrheal patients, with an exploratory uncorrected association between ST1 positivity and chronic diarrhea. This suggests that the link between ST1 and chronic diarrhea may be driven by the genetic heterogeneity of isolates, and the underlying mechanisms need to be further clarified by subsequent functional studies and large-scale cohort studies.

### 4.1 Study limitations

Several limitations of this study should be acknowledged. First, it was a single-center study with a relatively small sample size, which may introduce selection bias and restrict generalizability to the Shanghai population. Second, only human fecal specimens were collected, lacking synchronous sampling of animal reservoirs or environmental matrices, preventing identification of infection sources and transmission routes. Third, a single fecal specimen per participant was collected, unable to account for intermittent parasite shedding or monitor the correlation between detection and symptom progression. Fourth, this study is an observational case-control study, and no synchronous functional experiments were performed; therefore, causal inference cannot be drawn from the observed association between *Blastocystis* detection and diarrheal symptoms. Fifth, the statistical power for the ST1-chronic diarrhea subgroup analysis was only moderate (62.3%) due to the limited number of ST1-positive isolates. Although the observed association was statistically significant, this finding should be interpreted as exploratory and requires confirmation in larger cohorts with sufficient sample size. Sixth, the present analysis did not incorporate data on other common enteropathogens. Therefore, the potential confounding effect of co-infection on the observed association between *Blastocystis* and diarrheal symptoms cannot be excluded, and definitive conclusions regarding the independent role of *Blastocystis* in diarrheal symptoms cannot be drawn. Finally, the case group only included cases of acute and chronic diarrhea and the definition of chronic diarrhea (≥ 4 weeks) was based on WHO criteria; different definitions may limit cross-study comparability. Future investigations should use multi-center, large-scale prospective cohort designs, with synchronous sampling of human, animal, and environmental specimens, combined with high-resolution whole-genome sequencing and functional assays to clarify *Blastocystis*’ clinical relevance, transmission dynamics, and zoonotic potential.

## 5. Conclusion

In summary, this study systematically characterized the detection rate, subtype distribution, and genetic characteristics of *Blastocystis* among diarrheal outpatients in Shanghai, providing important baseline molecular epidemiological data for this enteric protozoan in the region. Our findings showed that *Blastocystis* detection was significantly correlated with diarrheal symptoms in the study cohort, with ST3 as the dominant circulating subtype. ST1 isolates were mainly detected in diarrheal patients, with high intrasubtype genetic heterogeneity, and an exploratory uncorrected association was observed between ST1 detection and chronic diarrhea. These results provide basic epidemiological data for further exploring the clinical correlation of *Blastocystis*, and offer reference for the optimization of clinical auxiliary screening and epidemiological surveillance of *Blastocystis* in the region.

## Supporting information

S1 Table Univariate logistic regression analysis of factors correlated with *Blastocystis* detection in the total cohort (n = 370). (DOCX)

S2 Table Univariate logistic regression analysis of factors correlated with *Blastocystis* detection in the non-diarrheal control group (n = 150). (DOCX)
