# Supplementary material for: Molecular detection and subtype characterization of Blastocystis in diarrheal outpatients in Shanghai, China: A case-control study
Source: PLoS Negl Trop Dis. 2026 Jun 26;20(6):e0014461. doi: 10.1371/journal.pntd.0014461 (PMC13308841; doi:10.1371/journal.pntd.0014461)
Supplement: S1 Table — (DOCX) [file pntd.0014461.s001.docx]

### S1 Table. Univariate logistic regression analysis of factors correlated with *Blastocystis* detection in the total cohort (n=370)

| **Factors** | **Subgroup** | **Total No.** | Blastocystis **Positive n** | Blastocystis **Negative n** | **Crude OR (95%CI)** | P **value** |
| --- | --- | --- | --- | --- | --- | --- |
| **Clinical phenotype** | Non-diarrheal (Ref) | 150 | 6 | 144 | 1.00 (Reference) | – |
|  | Acute diarrhea | 176 | 25 | 151 | 3.96 (1.58–9.91) | 0.003 |
|  | Chronic diarrhea | 44 | 12 | 32 | 9.17 (3.34–25.18) | <0.001 |
| **Age group** | 19–60 years (Ref) | 129 | 11 | 118 | 1.00 (Reference) | – |
|  | <6 years | 145 | 16 | 129 | 1.18 (0.50–2.79) | 0.709 |
|  | 7–18 years | 59 | 11 | 48 | 2.46 (0.99–6.10) | 0.052 |
|  | >60 years | 37 | 5 | 32 | 1.45 (0.44–4.77) | 0.541 |
| **Sex** | Male (Ref) | 185 | 18 | 167 | 1.00 (Reference) | – |
|  | Female | 185 | 25 | 160 | 1.43 (0.75–2.73) | 0.278 |
| **Residential region** | Urban (Ref) | 43 | 7 | 36 | 1.00 (Reference) | – |
|  | Suburban | 327 | 36 | 291 | 1.36 (0.58–3.17) | 0.481 |
