# Supplementary material for: Molecular detection and subtype characterization of Blastocystis in diarrheal outpatients in Shanghai, China: A case-control study
Source: PLoS Negl Trop Dis. 2026 Jun 26;20(6):e0014461. doi: 10.1371/journal.pntd.0014461 (PMC13308841; doi:10.1371/journal.pntd.0014461)
Supplement: S2 Table — (DOCX) [file pntd.0014461.s002.docx]

**S2 Table. Univariate logistic regression analysis of factors correlated with Blastocystis detection in the non-diarrheal control group (n=150)**

| **Factors** | **Subgroup** | **Total No.** | **Positive n** | **Negative n** | **Crude OR (95%CI)** | **P value** |
| --- | --- | --- | --- | --- | --- | --- |
| **Age group** | 19–60 years (Ref) | 54 | 2 | 52 | 1.00 | – |
|  | <6 years | 57 | 2 | 55 | 0.98 (0.13–7.27) | 0.984 |
|  | 7–18 years | 24 | 1 | 23 | 1.06 (0.10–11.50) | 0.960 |
|  | >60 years | 15 | 1 | 14 | 1.89 (0.18–20.30) | 0.593 |
| **Sex** | Male (Ref) | 68 | 1 | 67 | 1.00 | – |
|  | Female | 82 | 5 | 77 | 4.31 (0.50–37.02) | 0.186 |
| **Residential region** | Urban (Ref) | 12 | 1 | 11 | 1.00 | – |
|  | Suburban | 138 | 5 | 133 | 0.41 (0.04–4.00) | 0.440 |
