# Supplementary material for: Molecular detection and subtype characterization of Blastocystis in diarrheal outpatients in Shanghai, China: A case-control study
Source: PLoS Negl Trop Dis. 2026 Jun 26;20(6):e0014461. doi: 10.1371/journal.pntd.0014461 (PMC13308841; doi:10.1371/journal.pntd.0014461)
Supplement: Attachment — Submitted filename: Comments.pdf [file pntd.0014461.s003.pdf]

Manuscript: PNTD-D-26-00756 — *Molecular Detection and Subtype Characterization of* Blastocystis *in Diarrheal Outpatients in Shanghai, China: A Prospective Case-Control Study*

Recommendation: Major Revisions

Summary

Zhao and colleagues report a single-center study of *Blastocystis* prevalence and subtype distribution among 220 outpatients with diarrhea and 150 non-diarrheal controls recruited at a Shanghai tertiary hospital between June 2023 and March 2026. The work fills a regional gap in Chinese *Blastocystis* molecular epidemiology, and the underlying molecular methodology is sound. However, several substantive issues need to be addressed before the manuscript can be considered for publication.

Major Concerns

1. **Citation integrity.** Reference [28] (Zhao et al., 2014, *Appl Environ Microbiol*) is cited in the Discussion to support a comparison of Wenzhou *Blastocystis* prevalence (8.8% vs 2.0%), but that paper concerns *Enterocytozoon bieneusi* in pigs. The relevant Wenzhou pediatric *Blastocystis* paper is [23], which is correctly cited elsewhere for the same figure. A complete reference audit is required. The inline citation "(Stensvold et al., 2013)" at line 171 also breaks the numeric citation style.
2. **Statistical interpretation of the ST1–chronic-diarrhea association.** This finding is highlighted in the Abstract, Results, Discussion, and Conclusion as a "significant statistical association." Yet it rests on a Fisher's exact test of a 2×2 table with cell counts of 3, 22, 6, 6, without correction for the multiple subtype × phenotype × demographic

comparisons performed. Authors must either (a) apply a multiple-testing correction (e.g., Benjamini–Hochberg across all subtype comparisons) and report adjusted P-values, or (b) explicitly reframe the result as exploratory and hypothesis-generating throughout, including in the Abstract and Conclusion.

3. **Sample size and design transparency.** The study is labeled "case-control," but the recruitment frames for the control group are not described. Where were the 150 non-diarrheal "healthy individuals" identified (in the community, among employees, among outpatient companions, or at screening clinics)? How was the absence of diarrhea verified? Was "age- and sex-frequency matching" implemented prospectively (matched recruitment) or claimed post hoc? An a priori sample-size calculation is acknowledged to be absent. Please add a post hoc power discussion for the primary case/control comparison and for the ST1 subgroup analysis.
4. **Co-infection.** Given the central claim of an association between *Blastocystis* and diarrhea, the absence of concurrent screening for routine viral, bacterial, and other parasitic enteropathogens is a significant confounder, as noted in Limitation 6. Tertiary hospital outpatients with diarrhea typically undergo some microbiological testing. Please report any available co-infection data (even partial), or explicitly explain why such data could not be retrieved retrospectively.
5. **Genetic diversity comparison.** The claim that ST1 (29 SNPs across 10 isolates) is more genetically diverse than ST3 (3 SNPs across 33 isolates) requires a normalized metric — e.g., nucleotide diversity π, segregating sites per site, or Watterson's θ — that accounts for sample size and sequence length, because raw SNP counts are not directly comparable.

6. **Figure 1 methodology.** The "PubMed knowledge network" is introduced in the Introduction without a description of its construction (search terms, date range, software, edge-weighting rules, and semantic relationship coding). Please either provide complete methods in a supplementary file, replace it with a cited published network, or move it to the supplement.
7. **Persistent diarrhea.** WHO classifies diarrhea lasting 14 days to <4 weeks as persistent. The Methods define only acute (<14 days) and chronic (≥4 weeks). Please specify whether persistent cases were excluded, reclassified, or absent from the cohort.

Minor Concerns

1. **Hypothesis framing.** The Introduction lacks a clearly stated hypothesis. A 1–2-sentence predictive hypothesis would sharpen the rationale.
2. **Section title.** Section 3.4 reads "Phylogenetic"; please complete it to "Phylogenetic analysis."
3. **Grammar.** Several sentences are incomplete. For example, lines 86–87 ("systematic investigations into the clinical features and zoonotic risk of *Blastocystis* in large, highly mobile urban populations") lack a main verb.
4. **"Effective matching" claim** (Table 1 footnote). P > 0.05 indicates non-significant differences, not equivalence. Reword as "no statistically significant difference."
5. **Funding source consistency.** All four funding streams are philosophy and social sciences awards. Please briefly explain how these supported a molecular parasitology study, or note this in the acknowledgments.

6. **Use of "fold-increase" language.** "4.8-fold increased infection risk" (line 254) is derived from prevalence ratios and should be reported with its 95% CI, or replaced with the adjusted OR from the logistic regression, for greater precision.
7. **Sequence count.** 13 GenBank accessions are deposited for 43 positive amplicons; please confirm that these represent unique sequences (as implied) and explicitly state this in section 2.6.
8. **Abstract conclusion overstatement.** "Fills a critical regional data gap" is editorial; please soften.
