# Supplementary material for: Molecular detection and subtype characterization of Blastocystis in diarrheal outpatients in Shanghai, China: A case-control study
Source: PLoS Negl Trop Dis. 2026 Jun 26;20(6):e0014461. doi: 10.1371/journal.pntd.0014461 (PMC13308841; doi:10.1371/journal.pntd.0014461)
Supplement: Attachment — Submitted filename: Reviewer 2 Report.pdf [file pntd.0014461.s005.pdf]

Reviewer 2 Report

Comments to the Editor:

The authors have submitted a revised manuscript and provided detailed explanations for their modifications. Based on the "Response to Reviewers" section provided, the authors have systematically and adequately addressed all major and minor concerns I raised.

Evaluation of Revisions:

Major Concerns:

1. **Citation Integrity:** The authors conducted a comprehensive audit of references and corrected prior citation errors.
2. **Statistical Interpretation:** The association between ST1 and chronic diarrhea has been appropriately framed as an exploratory, hypothesis-generating finding throughout the manuscript, which is the appropriate course of action given the absence of multiple-testing corrections.
3. **Study Design Transparency:** The authors clarified the retrospective recruitment of the control group, described the post hoc matching process, and provided the requested post hoc power analysis.
4. **Co-infection Confounders:** The authors provided a sound methodological justification for excluding partial co-infection data from the main text. Furthermore, they appropriately strengthened the limitation statement to explicitly acknowledge the absence of comprehensive co-infection screening.

5. **Genetic Diversity:** Standardized metrics (nucleotide diversity, Watterson's θ, and segregating sites per site) were calculated and included in the results to enable a direct and proper comparison of genetic diversity.
6. **Methodology of Figure 1:** The authors chose to remove the "PubMed knowledge network" figure entirely, which effectively resolves the methodological concern without affecting the study's primary results.
7. **Persistent Diarrhea:** The authors clarified their adherence to WHO criteria and confirmed that the four patients with persistent diarrhea (lasting 14 days to less than 4 weeks) were excluded from the cohort.

Minor Concerns:

All minor issues have been fully and satisfactorily resolved. This includes adding a clear predictive hypothesis, correcting section titles and grammatical errors, rewording statistical claims, clarifying funding sources, using adjusted odds ratios, and softening editorial statements in the abstract.

Recommendation:

The authors have thoroughly and appropriately addressed all critical issues raised in the previous review round. The revisions are highly satisfactory, and no major revisions are strictly necessary from my perspective. Therefore, I recommend that the manuscript be accepted or handled with minor editorial revisions. The editorial team can proceed with the final decision and formatting directly, without returning the manuscript to me for another round of review.
