# Supplementary material for: Molecular detection and subtype characterization of Blastocystis in diarrheal outpatients in Shanghai, China: A case-control study
Source: PLoS Negl Trop Dis. 2026 Jun 26;20(6):e0014461. doi: 10.1371/journal.pntd.0014461 (PMC13308841; doi:10.1371/journal.pntd.0014461)
Supplement: Attachment — Submitted filename: Response to Reviewers.docx [file pntd.0014461.s004.docx]

**Response to Reviewers**

**Reviewer #1:**

The authors have done a single centre, case control study with focus on describing the prevalence rate, molecular epidemiology and clinical correlates of Blastocystis species in a localized region of China. The study adds to the limited but growing literature on the clinical significance of *Blastocystis* species. While the study is excellently done there are few suggestions by addressing them can improve the overall quality of the article.

**To Reviewer #1:** We sincerely appreciate your time, review, and constructive feedback on our manuscript. Your comments have enhanced the scientific rigor, clarity, and quality of this work and guided our future research. We've addressed all concerns and revised the manuscript as requested. If you have more comments or suggestions, please tell us. We'll happily make further revisions to meet the journal's publication standards. Thank you again for your contribution to improving this study.

**Q1.** Lines 90-91, “To date, few comprehensive molecular epidemiological study has characterized *Blastocystis* prevalence, subtype distribution, and clinical associations in diarrheal patients in Shanghai”, the sentence should be grammatically corrected.

**To Q1**. We apologize for the grammatical error. We have corrected this sentence as follows: “**To date, few comprehensive molecular epidemiological studies have characterized *Blastocystis* prevalence, subtype distribution, and clinical associations in diarrheal patients in Shanghai.” (Line 100)**

**Q2.** In line 96, the authors have stated that one of the objectives of the study is to provide evidence for optimized local gastrointestinal disease management and surveillance, however the disease doesn’t provide any objective evidence related to it in the results or discussion section. Hence, in my opinion, the authors may consider the removal of this objective.

**To Q2.** “, and provide evidence for optimized local gastrointestinal disease management and surveillance” has been deleted. **(Line 108)**

**Q3.** In Lines 253-257, the authors have compared their prevalence rates with other studies however, the patient population is different from the one used in their study. For e.g., in Reference 26, the 33.3% prevalence rate is in patients with IBS. The authors should categorically compare their rates with the studies which have similar patient population as in their study and if not, should mention briefly the patient population used in the comparator study.

**To Q3.** We apologize for the lack of clarity in the original comparison. We have revised this section to explicitly state the patient population of each comparator study. **(Lines 285-297)**

**Q4.** Reference formatting should be made uniform. In certain references, “capitalise each word” function is used whereas in others, “sentence case” is used.

**To Q4.** We have standardized all references to sentence case (only the first word of the title, the first word of the subtitle, and proper nouns are capitalized) in accordance with the PLOS Neglected Tropical Diseases reference style. A complete reference audit has been performed to ensure consistency.

**Reviewer #2:**

**Manuscript:** PNTD-D-26-00756 — *Molecular Detection and Subtype Characterization of* Blastocystis *in Diarrheal Outpatients in Shanghai, China: A Prospective Case-Control Study*

**Recommendation:** Major Revisions

**Summary**

Zhao and colleagues report a single-center study of *Blastocystis* prevalence and subtype distribution among 220 outpatients with diarrhea and 150 non-diarrheal controls recruited at a Shanghai tertiary hospital between June 2023 and March 2026. The work fills a regional gap in Chinese *Blastocystis* molecular epidemiology, and the underlying molecular methodology is sound. However, several substantive issues need to be addressed before the manuscript can be considered for publication.

**To Reviewer #2:** We sincerely appreciate your valuable time, rigorous review, and constructive feedback on our manuscript. Your insightful comments have not only significantly enhanced the scientific rigor, clarity, and overall quality of this work but also provided invaluable guidance for our future research endeavors. We have carefully addressed all the concerns raised and made comprehensive revisions to the manuscript as requested. Should you have any additional comments or suggestions, please do not hesitate to contact us. We will be more than happy to make further revisions to meet the publication standards of the journal. Thank you again for your invaluable contribution to improving this study.

**Major Concerns**

**Major Q1. Citation integrity.** Reference [28] (Zhao et al., 2014, *Appl Environ Microbiol*) is cited in the Discussion to support a comparison of Wenzhou *Blastocystis* prevalence (8.8% vs 2.0%), but that paper concerns *Enterocytozoon bieneusi* in pigs. The relevant Wenzhou pediatric *Blastocystis* paper is [23], which is correctly cited elsewhere for the same figure. A complete reference audit is required. The inline citation "(Stensvold et al., 2013)" at line 171 also breaks the numeric citation style.

**To Major Q1.** We sincerely apologize for these citation errors, which were caused by our carelessness. A comprehensive reference audit has been carried out to verify the accuracy of all citations in the manuscript, and the incorrect citations have been corrected. **(Lines 149,183,288)**

**Major Q2. Statistical interpretation of the ST1–chronic-diarrhea association.** This finding is highlighted in the Abstract, Results, Discussion, and Conclusion as a "significant statistical association." Yet it rests on a Fisher's exact test of a 2×2 table with cell counts of 3, 22, 6, 6, without correction for the multiple subtype × phenotype × demographic comparisons performed. Authors must either (a) apply a multiple-testing correction (e.g., Benjamini–Hochberg across all subtype comparisons) and report adjusted P-values, or (b) explicitly reframe the result as exploratory and hypothesis-generating throughout, including in the Abstract and Conclusion.

**To Major Q2.** Thank you for this critical statistical comment. We agree that the uncorrected P-value for the ST1-chronic diarrhea association should be interpreted with caution given the multiple comparisons performed. We have chosen option (b) and have explicitly reframed this finding as exploratory and hypothesis-generating throughout the manuscript, including in the Abstract, Results, Discussion, and Conclusion. **(Lines 27,28,31-33,252,253,367,368,399,400)**

**Major Q3. Sample size and design transparency.** The study is labeled "case-control," but the recruitment frames for the control group are not described. Where were the 150 nondiarrheal "healthy individuals" identified (in the community, among employees, among outpatient companions, or at screening clinics)? How was the absence of diarrhea verified? Was "age- and sex-frequency matching" implemented prospectively (matched recruitment) or claimed post hoc? An a priori sample-size calculation is acknowledged to be absent. Please add a post hoc power discussion for the primary case/control comparison and for the ST1 subgroup analysis.

**To Major Q3:** Thank you for this important comment on study design transparency. We have added detailed information about the control group recruitment and matching method to the Materials and Methods section **(Lines 121-124)**. **The control group was recruited retrospectively after the completion of case recruitment. 73 controls were from the physical examination center of the same hospital, and 77 controls (all <18 years old) were from 4 kindergartens, primary schools, junior high schools, and high schools in the same residential area as the hospital. Control recruitment was conducted as part of our social science project on enteric protozoan infection risk assessment, which included health education activities on intestinal parasitic diseases. The absence of diarrhea, abdominal discomfort, or other gastrointestinal symptoms in the past three months was verified through structured questionnaires administered to all control participants.**

Sample size calculation was not performed prior to study initiation due to the exploratory and observational design of this investigation. **Age- and sex-frequency matching was performed post hoc to ensure comparable distribution of these variables between the case and control groups.** Meanwhile, according to your suggestions, we have added detailed post hoc power analysis results to the end of the 2.8 Statistical analysis section in the revised manuscript **(Lines 208-215).**

**Major Q4. Co-infection.** Given the central claim of an association between *Blastocystis* and diarrhea, the absence of concurrent screening for routine viral, bacterial, and other parasitic enteropathogens is a significant confounder, as noted in Limitation 6. Tertiary hospital outpatients with diarrhea typically undergo some microbiological testing. Please report any available co-infection data (even partial), or explicitly explain why such data could not be retrieved retrospectively.

**To Major Q4.** Thank you for this critical and insightful comment regarding co-infection as a potential confounder. We fully acknowledge that concurrent infection with other enteropathogens is an important limitation of this study. We have comprehensively reviewed all available clinical laboratory records for the 220 diarrheal patients enrolled and provide a complete summary of all retrievable co-infection data below for your evaluation.

**Enteric virus testing:** Nearly all acute diarrhea patients (n=176) underwent routine clinical testing for three common enteric viruses: group A rotavirus (RVA), adenovirus, and norovirus GII (NoV GII). A total of 40 (22.7%) patients tested positive for at least one virus, including: 12 with rotavirus mono-infection; 8 with adenovirus mono-infection; 9 with norovirus mono-infection; 5 with rotavirus-adenovirus co-infection; 2 with rotavirus-norovirus co-infection; 1 with norovirus-adenovirus co-infection; 3 with triple infection of all three viruses.

**Bacterial pathogens:** No comprehensive screening for bacterial enteropathogens was performed as part of this study. Retrospective retrieval of bacterial culture results was not possible due to incomplete electronic medical record documentation for outpatient visits during the study period.

**Meanwhile, our laboratory has completed the testing for other intestinal protozoa.** A subset of 147 diarrheal patients was additionally tested for *Enterocytozoon bieneusi* and *Cryptosporidium* spp. using the molecular methods. Twenty-one (14.3%) patients were positive for *E. bieneusi* (Ke J, Sun L, Yu Q, Yu X, Zhang Z, Jiang A, Peng X, Zhang J, Jiang F, Jiang Y, Zhou H, Zhao W. PCR-based detection of Enterocytozoon bieneusi in diarrheic patients from Guangdong, Shandong, Shanghai, and Zhejiang Provinces, China: a study on prevalence and genotypic characteristics. Front Cell Infect Microbiol. 2026 Jan 9;15:1728500. doi: 10.3389/fcimb.2025.1728500. PMID: 41586313; PMCID: PMC12827597.), while no *Cryptosporidium* infections were detected in this subset (The data have not been published).

After careful methodological consideration, we have decided not to add these co-infection data to the main text or modify the original results description for the following scientifically justified reasons:

1) The enteric virus testing was only performed in acute diarrhea patients, while chronic diarrhea patients did not receive routine viral testing, resulting in incomplete and non-uniform data across the entire study cohort.
2) The *E. bieneusi* and *Cryptosporidium* testing was only conducted in a subset of patients (147/220), which would introduce significant selection bias if included in the primary analysis.
3) The primary objective of this study was to characterize the molecular epidemiology and clinical associations of Blastocystis in Shanghai, not to systematically evaluate the impact of co-infections. Including partial co-infection data without a corresponding statistical analysis of their confounding effects would be methodologically unsound and could lead to misleading interpretations.
4) We have retained and slightly strengthened the original limitation statement in the Discussion section, which explicitly acknowledges: "This study did not perform synchronous comprehensive screening for other common enteropathogens, which cannot exclude the potential confounding effect of co-infection on the observed association between Blastocystis and diarrheal symptoms." We believe this accurately reflects the study's limitations without overinterpreting incomplete data. **(Lines 380-383)**

*We appreciate your bringing this important issue to our attention. We have provided all available co-infection data in this response for your full evaluation and believe that retaining the original manuscript structure while clearly acknowledging the limitation of incomplete co-infection screening represents the most scientifically rigorous approach. Should you have any additional concerns or suggestions, please do not hesitate to let us know.*

**Major** Q5. **Genetic diversity comparison.** The claim that ST1 (29 SNPs across 10 isolates) is more genetically diverse than ST3 (3 SNPs across 33 isolates) requires a normalized metric-e.g., nucleotide diversity π, segregating sites per site, or Watterson's θ-that accounts for sample size and sequence length, because raw SNP counts are not directly comparable.

**To Major Q5.** Thank you for this critical and methodologically rigorous comment. We fully agree that raw SNP counts are not directly comparable across subtypes with different sample sizes and sequence lengths. We have calculated three standardized genetic diversity metrics (nucleotide diversity π, Watterson's θ, and segregating sites per site) for both ST1 and ST3 isolates using DnaSP 6.0 software, and have added these results to the Results section along with a new table summarizing the normalized indices. Meanwhile, we have added relevant methodological content. **(Lines 186-190; 254-260; Table 4)**

**Major Q6. Figure 1 methodology.** The "PubMed knowledge network" is introduced in the Introduction without a description of its construction (search terms, date range, software, edge-weighting rules, and semantic relationship coding). Please either provide complete methods in a supplementary file, replace it with a cited published network, or move it to the supplement.

**To Major Q6.** Thank you for this important comment. We fully acknowledge that the "PubMed knowledge network" figure lacked adequate methodological description. We have completely removed Figure 1 and all corresponding references from the revised manuscript. This figure was only a visual summary of existing literature and did not present any original data or core findings of our study. Its removal does not affect the validity of our primary results. We apologize for this oversight and confirm that all remaining figures and tables are accompanied by complete methodological descriptions.

**Major Q7. Persistent diarrhea.** WHO classifies diarrhea lasting 14 days to <4 weeks as persistent. The Methods define only acute (<14 days) and chronic (≥4 weeks). Please specify whether persistent cases were excluded, reclassified, or absent from the cohort.

**To Major Q7.** This study adhered to the WHO classification criteria for diarrhea, and only 4 patients met the definition of persistent diarrhea lasting "14 days to <4 weeks," thus being excluded. Consequently, the study included only patients with acute (<14 days) or chronic (≥4 weeks) diarrhea.

**Minor Concerns**

**Minor** Q1. **Hypothesis framing.** The Introduction lacks a clearly stated hypothesis. A 1–2-sentence predictive hypothesis would sharpen the rationale.

**To Minor Q1.** Thank you for this constructive suggestion that has significantly sharpened our study rationale. We have added a clear 2-sentence predictive hypothesis at the end of the Introduction section, which naturally connects the previously identified research gaps to our study objectives. **(Lines 102-108)**

**MinorQ2.** **Section title.** Section 3.4 reads "Phylogenetic"; please complete it to "Phylogenetic analysis."

**To Minor Q2.** We have corrected the section title as suggested: **3.4 Phylogenetic analysis. **(Line 273)****

**Minor** Q3. **Grammar.** Several sentences are incomplete. For example, lines 86–87 ("systematic investigations into the clinical features and zoonotic risk of *Blastocystis* in large, highly mobile urban populations") lack a main verb.

****To** Minor Q3**: We have corrected this incomplete sentence and have reviewed the entire manuscript for additional grammatical errors. **(Line 97, 100)**

**Minor Q**4. **"Effective matching" claim** (Table 1 footnote). P > 0.05 indicates non-significant differences, not equivalence. Reword as "no statistically significant difference."

**To Minor Q4.** We have revised the Table 1 footnote as suggested. **P > 0.05 indicates no statistically significant difference in baseline characteristics between groups.**

**Minor Q**5. **Funding source consistency.** All four funding streams are philosophy and social sciences awards. Please briefly explain how these supported a molecular parasitology study, or note this in the acknowledgments.

**To Minor Q5**: These social science projects centered on intestinal protozoa infection risk assessment and public health intervention among diverse populations, supporting community health education, field investigation coordination, data sorting, manuscript composition and article publication fees. Laboratory testing work was funded by the Basic Scientific Research Project of Wenzhou (Y2023070) and internal research funds from Wenzhou Medical University. The funders exerted no influence on study design, data acquisition and analysis, manuscript drafting or publication decision.

**Minor Q6. Use of "fold-increase" language.** "4.8-fold increased infection risk" (line 254) is derived from prevalence ratios and should be reported with its 95% CI, or replaced with the adjusted OR from the logistic regression, for greater precision.

**To Minor Q6.** We have replaced the "fold-increase" language with the adjusted OR and 95% CI from the multivariate logistic regression, as suggested: with **an adjusted OR of 4.78 (95% CI: 1.92–11.91, P < 0.001). **(Line 284,285)****

**Minor Q7. Sequence count.** 13 GenBank accessions are deposited for 43 positive amplicons; please confirm that these represent unique sequences (as implied) and explicitly state this in section 2.6.

**To Minor Q7.** “**representative” has been added**. **(Line183)**

**Minor Q8** **Abstract conclusion overstatement.** "Fills a critical regional data gap" is editorial; please soften.

**To Minor Q8**: We have softened this statement in the Abstract and Conclusion sections. **(Lines 33,34,395,396)**
